# Volume entropy for modeling information flow in a brain graph

**DOI:** 10.1038/s41598-018-36339-7

**Published:** 2019-01-22

**Authors:** Hyekyoung Lee, Eunkyung Kim, Seunggyun Ha, Hyejin Kang, Youngmin Huh, Youngjo Lee, Seonhee Lim, Dong Soo Lee

**Affiliations:** 1 Biomedical Research Institute, Seoul National University Hospital, Seoul, South Korea; 2 Department of Nuclear Medicine, Seoul National University College of Medicine, Seoul, South Korea; 3 Department of Rehabilitation Medicine, Seoul National University Hospital, Seoul, South Korea; 4 Data Science for Knowledge Creation Research Center, Seoul National University, Seoul, South Korea; 5 Interdisciplinary Program in Cognitive Science, Seoul National University, Seoul, South Korea; 6 Department of Statistics, College of Natural Sciences, Seoul National University, Seoul, South Korea; 7 Department of Molecular Medicine and Biopharmaceutical Sciences, Graduate School of Convergence Science and Technology, Seoul National University, Seoul, South Korea; 8 Department of Mathematical Sciences, Seoul National University, Seoul, South Korea; 9 Korea Brain Research Institute, Daegu, Republic of Korea; 10 BK21 Plus Global Translational Research on Molecular Medicine and Biopharmaceutical Sciences, Seoul National University, Seoul, South Korea

## Abstract

Brain regions send and receive information through neuronal connections in an efficient way. In this paper, we modelled the information propagation in brain networks by a generalized Markov system associated with a new edge-transition matrix, based on the assumption that information flows through brain networks forever. From this model, we derived new global and local network measures, called a volume entropy and the capacity of nodes and edges on FDG PET and resting-state functional MRI. Volume entropy of a metric graph, a global measure of information, measures the exponential growth rate of the number of network paths. Capacity of nodes and edges, a local measure of information, represents the stationary distribution of information propagation in brain networks. On the resting-state functional MRI of healthy normal subjects, these measures revealed that volume entropy was significantly negatively correlated to the aging and capacities of specific brain nodes and edges underpinned which brain nodes or edges contributed these aging-related changes.

## Introduction

Brain is typically represented by a complex network, where its regions are functionally connected to each other^1^. Brain’s functional connections are inferred by the interregional correlation of physio-molecular signals between brain regions on brain imaging data such as functional magnetic resonance imaging (fMRI) or positron emission tomography (PET)^2–7^. The functional brain network is considered as an efficient and versatile system. The information spreads rapidly throughout the whole brain. In addition, there are sufficient alternative paths between brain regions from a geometric point of view. This property is referred to being locally efficient, which is often found in a modular network. Efficiency and versatility of brain networks have been quantified by the complex graph theoretic measures such as global and local efficiencies, characteristic path length, and clustering coefficient^8–10^. Many studies have reported the topological alterations of brain networks with normal aging or disease progression based on these complex graph measures^11–13^.

In this study, we propose a new entropy-based network measure, called a volume entropy, which is derived from an information propagation model^14^. The volume entropy is theoretically the topological entropy of the geodesic flow on a finite graph without terminal vertex. To estimate the volume entropy, we assume that the information flows through the edges on a network, and, if the time goes to infinity, the number of network paths of information flow will increase exponentially. We modelled the information propagation by a generalized Markov system (GMS) associated with a new edge-transition matrix^14^. The volume entropy measures the exponential growth rate of the number of network paths. The larger the volume entropy is, the more information flows on the graph. The stationary equation of the proposed GMS also provides the information capacity of nodes and edges as well as the direction of information flow on a graph. Therefore, the GMS modelling allowed us to derive both global and local network measures of information propagation on a network simultaneously, while the existing graph theoretic network measures were not derived from any consistent model of information exchanges of brain networks. Functional and spectral entropies were proposed as the entropy-based network measures of human brain^15,16^. They were based on the information entropy that measured the average negative logarithm of the probability distribution of a system in information theory^17^. Both of these entropies required a procedure to approximate the probability distribution of networks. However, this approximation procedure included the parameter selection for the probability distribution of a network, which was not justified yet.

On simulations, we compared the volume entropy of various artificial networks such as regular, small-world, random, scale-free, and hyperbolic networks. The results showed that the volume entropy distinguished the underlying graph topology and geometry better than the existing network measures such as global and local efficiencies as well as preexisting entropy-based measures. On human brain images, we measured the volume entropy and the capacities of brain’s nodes and edges of the brain graphs. On the resting state fMRI and PET obtained from 38 normal individuals between the ages of 20 s and 60 s, volume entropy revealed the change of volume entropy according to normal aging and the specific brain areas contributing these changes, which represented the aging-related decline of information flows in the brain.

## Results

### Generalized Markov system and volume entropy

**Figure 1:**
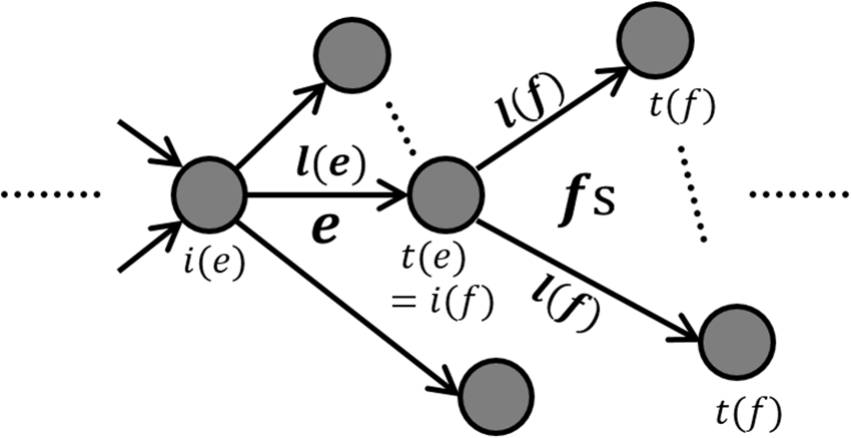
Paths from *e* to *f*s in the stationary equation, ${z}_{e}={\sum }_{f}\,{a}_{ef}{e}^{-hl(f)}{z}_{f}$.

Suppose that a weighted directed network is given with *p* nodes and *q* directed edges. We let *e* and *f* denote directed edges. The length of the edge *f* is denoted by *l*(*f*). We define a new edge-transition matrix $\,{\boldsymbol{L}}(h)=[{L}_{ef}]\in {{\mathbb{R}}}^{q\times q}$ by

$${L}_{ef}(h)={a}_{ef}{e}^{-hl(f)},$$

where *a*_*ef*_ is the usual edge-transition matrix, i.e, it is 1 if *e* is connected to *f* in the network, 0, otherwise, and *h* is a nonnegative constant^14^. We normalize the volume of the directed weighted network, say ${\sum }_{\forall e}\,l(e)=2.$ Then, the GMS associated to ***L***(*h*) is defined by

1 $$\,{{\boldsymbol{z}}}_{k}=L(h){{\boldsymbol{z}}}_{k+1},$$

for *h* > 0, *k* > 0, and $\,{{\boldsymbol{z}}}_{k}\in {{\mathbb{R}}}^{q}$. The dimension of ***z***_*k*_ is equal to the number *q* of directed edges in the network. The stationary equation of the GMS in (1) is written by ***z*** = ***L***(*h*) ***z***, where $\,{\boldsymbol{z}}(=[{z}_{e}])={{\boldsymbol{z}}}_{k}={{\boldsymbol{z}}}_{k+1}\in {{\mathbb{R}}}^{q}.$ If we rewrite the stationary equation for the edge *e*, the equation is as follows (see in Fig. 1):

2 $${z}_{e}=\sum _{f}\,{a}_{ef}{e}^{-hl(f)}{z}_{f}.$$

It implies that the number of paths in a network increases exponentially over time with the exponential growth rate *h*. The positive real number *h* that satisfies the stationary equation is referred to as the volume entropy and denoted by *h*_*vol*_. More detailed explanation of the volume entropy and toy example are in the method section.

### Volume entropy and aging

**Figure 2:**
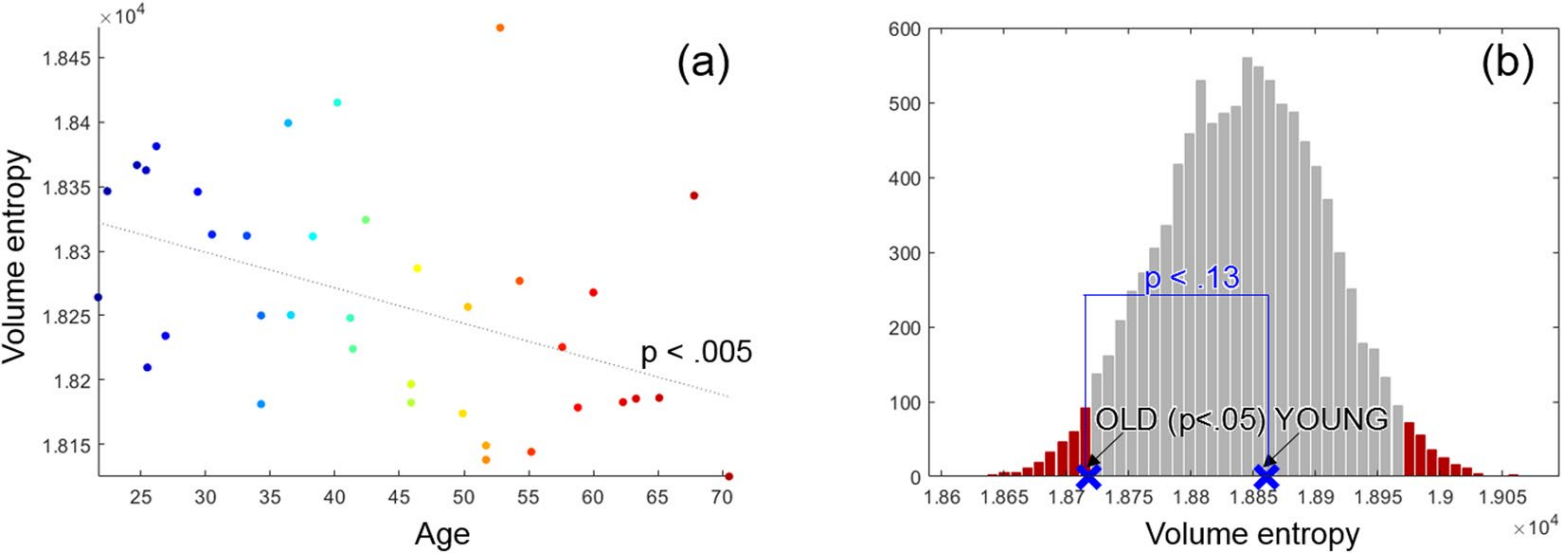
(**a**) Normalized volume entropy of 38 resting state functional networks with respect to age. The volume entropy was significantly negatively correlated with age (*p* < 0.005). (**b**) Normalized volume entropy of metabolic networks of the groups Y and O. The histogram showed the volume entropies of 5000 null networks constructed by permuted Y and O groups of the PET data set. The blue marker ‘X’ represented the volume entropy of the true metabolic networks, Y (right) and O (left). The volume entropy of O was significantly different from that of null networks (*p* < 0.05), but the volume entropy of Y was not. The difference of the volume entropy between Y and O was not significant, but showed the tendency of Y > O (*p* < 0.13).

We measured the volume entropy of the resting-state functional and metabolic brain networks obtained from the fMRI and PET data, respectively. The PET and fMRI data were simultaneously acquired from 38 healthy normal subjects (M/F: 19/18, mean age: 43.9 ± 13.9) from 20 s to 60 s using a Siemens Biograph mMR 3 T scanner. We constructed 38 functional networks of 38 subjects using the fMRI data and two metabolic networks of young (Y) and old (O) groups using the PET data. The groups Y and O were divided by whether the subjects were over 45 years old or not. The volume entropy and the age of fMRI-derived functional networks were negatively correlated (*p* < 0.005) (See in Fig. 2(a)). Y and O groups of PET-derived metabolic networks were compared using the null distribution made by performing 5000 permutations of Y and O groups. A graph constructed by permutation of Y and O was considered a null network, and 5000 null networks yielded the null distribution of the volume entropy as is shown as histogram in Fig. 2(b). The volume entropy of O was significantly smaller than that of null networks (*p* < 0.05), however, the volume entropy of Y was not (See the blue markers in Fig. 2(b)). The difference between Y and O was not significant, but showed the tendency that the volume entropy of Y was larger than that of O (*p* < 0.13). The correlation with ages and comparison between Y and O groups of functional and metabolic networks revealed that the volume entropy decreased with normal aging.

### Edge and node capacity of information flow

In the stationary equation of (2), *z* = [*z*_*e*_] is the normalized eigenvector of ***L***(*h*_*vol*_) with the largest eigenvalue 1. *z* has all entries of the same sign according to the Perron-Frobenius theorem. The sum of all *z*_*e*_ s is equal to one, i.e., ${\sum }_{e}\,{z}_{e}=1$. Thus, ***z*** = [*z*_*e*_] is the stationary distribution of the GMS associated to the edge-transition matrix ***L***(*h*_*vol*_).

We reshape the vector *z* into a matrix, $\,{\boldsymbol{\Pi }}=[{\pi }_{it}={z}_{e}]\in {{\mathbb{R}}}^{p\times p},$ where an edge *e* has the initial node *i*(*e*) = *i* and the terminal node *t*(*e*) = *t*, and *π*_*ii*_ = 0 (*i* = 1, …, *p*). Note that *p* is the number of nodes in a network. Since *π*_*it*_(= *z*_*e*_) is related to the number of paths in the graph that go through the edge *e* at the stationary state of the proposed system, we call it the edge capacity. The matrix **Π** is called an edge capacity matrix.

For a given oriented edge *e* from *i*(*e*) to *t*(*e*), we denote by $\bar{e}$ the oriented edge from $i(\bar{e})=t(e)=t$ to $t(\bar{e})=i(e)=i$. Note that for any *e* ∈ *E*, both *e* and $\bar{e}$ exist in the network. In the stationary equation (2), *z*_*e*_(= *π*_*it*_) is affected by the distance *l*(*f*) for all edges *f* s connected with the node *t*. On the other hand, ${z}_{\bar{e}}(\,={\pi }_{ti})$ is affected by the edge distances connected with the node *i*. Thus, *π*_*it*_ is different from *π*_*ti*_. The difference between *π*_*it*_ and *π*_*ti*_ is related to the imbalance of the connectivities of two nodes *i* and *t*. We define a node capacity by the difference between the inward and outward edge capacities of a node, estimated by ${\pi }_{i}={\sum }_{t}\,{\pi }_{ti}-{\sum }_{t}\,{\pi }_{it}$. If a node capacity is negative/positive, the outgoing edge capacities are larger/smaller than the incoming edge capacities.

### Edge and node capacities on a metabolic network

**Figure 3:**
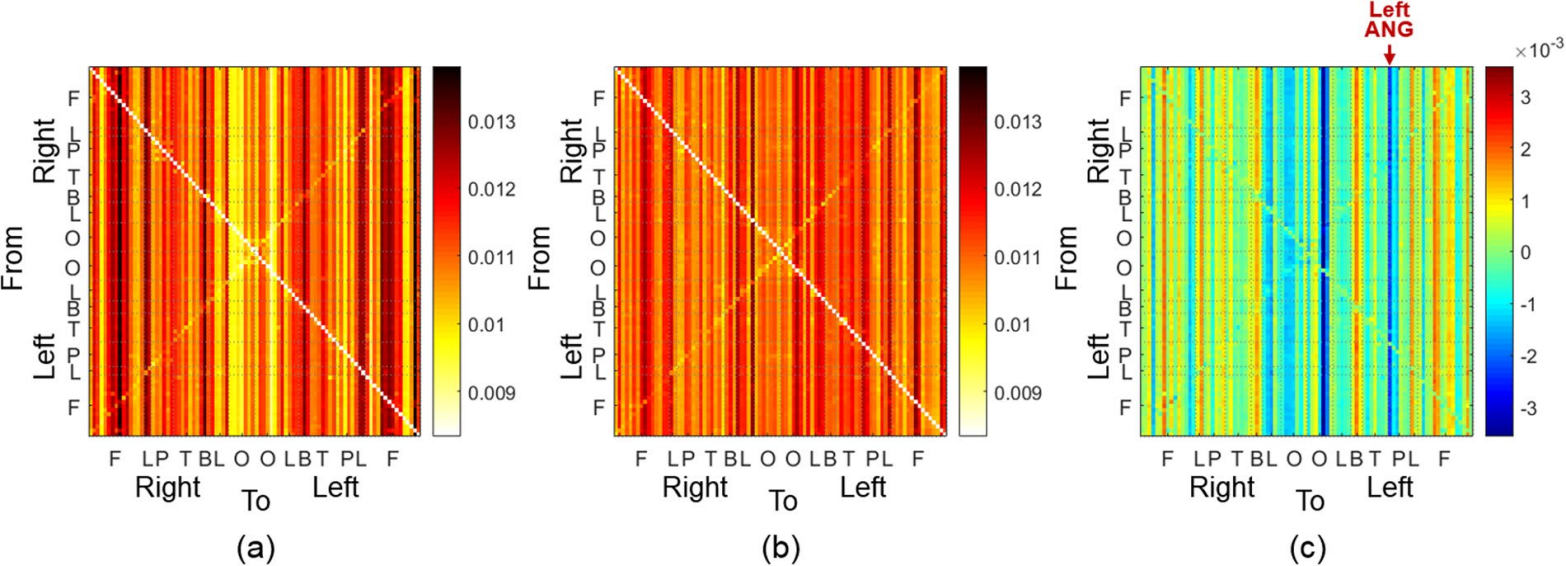
(**a,b**) Edge capacity matrices of the groups Y and O in PET. (**c**) Difference between the edge capacity matrices of Y and O. In the edge capacity matrix, the first 45 rows and columns corresponded to the right hemisphere and the last 45 rows and columns corresponded to the left hemisphere. F, L, P, T, B, L, and O represented frontal, limbic (cingulate cortex), parietal, temporal, basal ganglia, limbic (hippocampus and parahippocampal gyrus), and occipital lobes.

**Figure 4:**
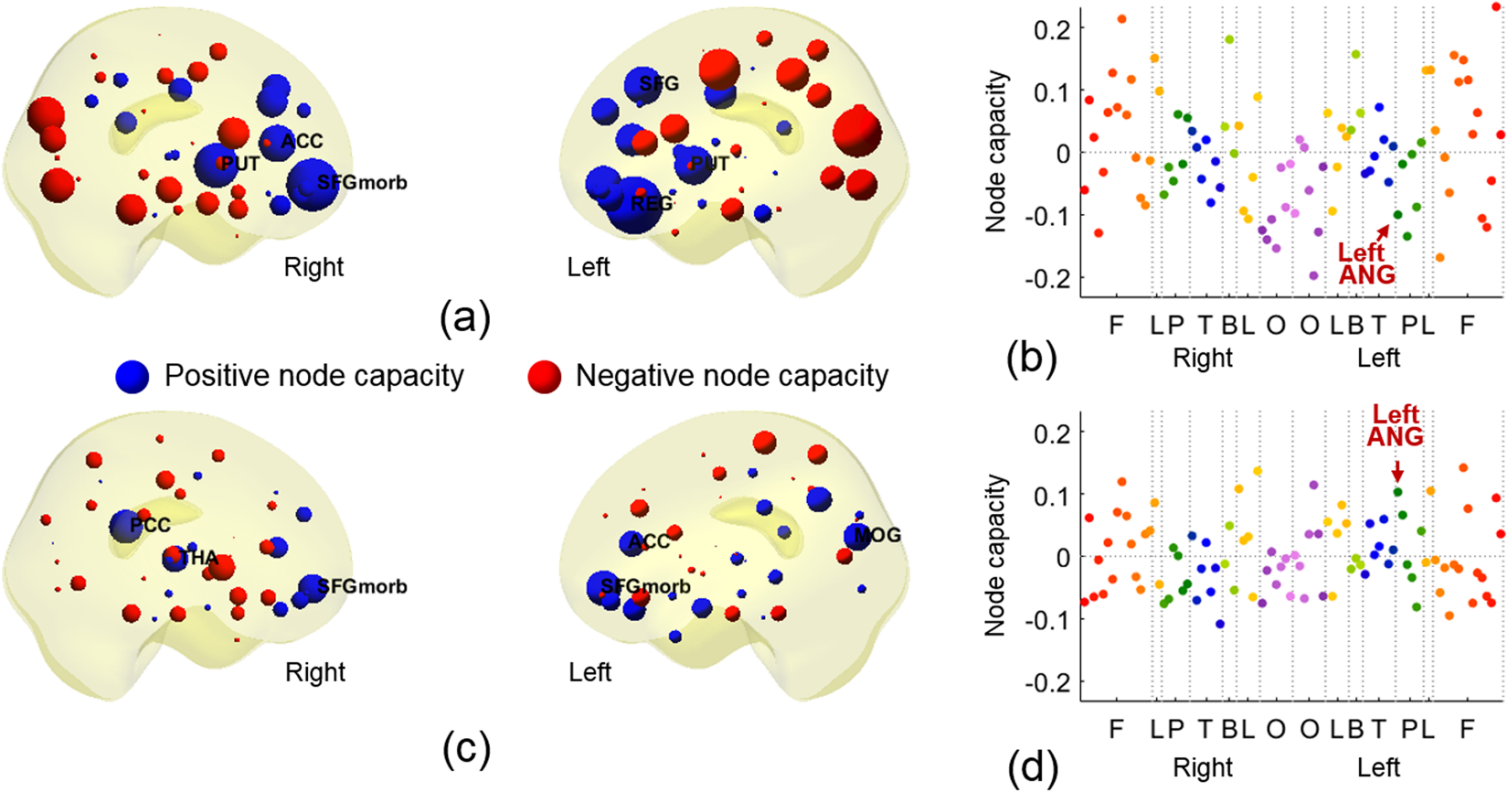
(**a,c**) Node capacities of Y and O obtained from the edge capacity matrices in Fig. 3(a,b). The size of a node was proportional to the absolute value of a node capacity. The color of a node was determined by the location of nodes. (**b,d**) Node capacities of Y and O in PET. The color of a dot represented the location of a node (more details in Sec. 1, Supplementary Material). The order of nodes was the same as the edge capacity matrix in Fig. 3(a,b). The red asterisk shows the significantly different brain region, left ANG between Y and O (*p* < 0.05, FDR-corrected).

Figures 3 and 4 showed the edge capacities and node capacities of the metabolic networks of the groups Y and O. The edge capacity matrices of Y and O were shown in Fig. 3(a,b), respectively. The difference between the edge capacity matrices of Y and O was shown in (c). The node capacities of Y and O were shown in Fig. 4(a–d), respectively. In the edge capacity matrix, the first 45 rows and columns were the nodes in the right hemisphere, and the last 45 rows and columns were in the left hemisphere. The nodes were sorted in the order of the frontal (F), limbic (L), parietal (P), temporal (T), basal ganglia (B), limbic (L), and occipital (O) lobes (more details in the Sec. 1 of the Supplementary Material). The (*i*, *t*)−th entry of the edge capacity matrix was the edge capacity directed from the node *i* to *t*. As the edge capacity decreased, the color of entry was changed from dark red to white as shown in the right colorbar. In the edge capacity matrices in Fig. 3(a,b), the entries of each column turned out to have similar color, which meant that the edges connected to the same terminal node had similar edge capacities. Figure 4(a,b) showed the node capacities of Y, and (c) and (d) showed that of O. In (a) and (c), the size of a node is proportional to the absolute value of a node capacity in (b) and (d), respectively. The positive node capacities were represented in blue color meaning larger incoming edge capacities and negative node capacities in red color meaning larger outgoing edge capacities. The positive and negative node capacities were represented by blue and red, respectively. In Fig. 4(b,d), the color of a point represented the location of a node: red and orange in F, green in P, blue in T, purple in O, yellow in L, and yellow-green in B (more details in the Sec. 1 of the Supplementary Material). In the edge capacity matrix of Y in Fig. 3(a), the edges were mainly directed to the medial orbital part of the superior frontal gyrus (SFGmorb) in the right hemisphere, bilateral putamen (PUT), left dorsolateral superior frontal gyrus (SFG), and left gyrus rectus (REG). In the edge capacity matrix of O in Fig. 3(b), the edges were mainly directed to bilateral SFGmorb, right thalamus (THA), right posterior cingulate cortex (PCC), and left middle occipital gyrus (MOG). Comparing edge capacity matrices and node capacity matrices, the terminal nodes of the edges with large capacity (such as in nodes of F and B in Y group or in nodes of F and O in O group) also had large node capacity shown as Fig. 4(a,c).

We performed 5000 permutations of Y and O and Wilcoxon rank sum test to find the difference between the edge capacities of Y and O in Fig. 3(c), that is to say the comparison between edge capacity matrices of Fig. 3(a,b). We could not find any edge showing larger capacity in Y than in O. In contrast, edge capacity of the left angular gyrus (ANG) was larger in O than as shown in the red arrow in Fig. 3(c) (*p* < 0.05, FDR-corrected). Correspondingly, the node capacity of the left ANG was larger in O than in Y as shown the red arrow in Fig. 4(b,d) (*p* < 0.05, FDR-corrected). This meant that the node capacity changed significantly with aging from negative node capacity in Y (red color in Y of Fig. 4(a) meaning larger outgoing edge capacity from the left ANG in Y) to positive node capacity in O (blue color in O of Fig. 4(c) meaning larger incoming edge capacity to the left ANG in O). However, in the pictures of node capacities of Y and O groups in Fig. 4(a,c), prominently incoming blue node capacities in the anterior regions of the brain looked larger in Y which came to be smaller blue or even smaller red (outgoing) nodes in O. And prominently outgoing red node capacities in the posterior regions of the brain looked larger in Y which came to be smaller red or even smaller blue (incoming) nodes in O. These changes did not achieve statistical significance after FDR-correction.

### Edge and node capacities on a functional network

The edge and node capacities of 38 functional networks obtained by fMRI data were shown in the Sec. 2 of the Supplementary Material. In the edge capacity matrices in Supplementary Fig. 2, networks of individuals showed variation which we could not summarize easily especially in terms of age-related changes, however, the entries of each column showed the similar colors, which meant that the edges connected to the same terminal nodes had similar edge capacities. This tendency of variation was also represented in the individual distribution of node capacities of functional networks in Supplementary Fig. 3.

**Figure 5:**
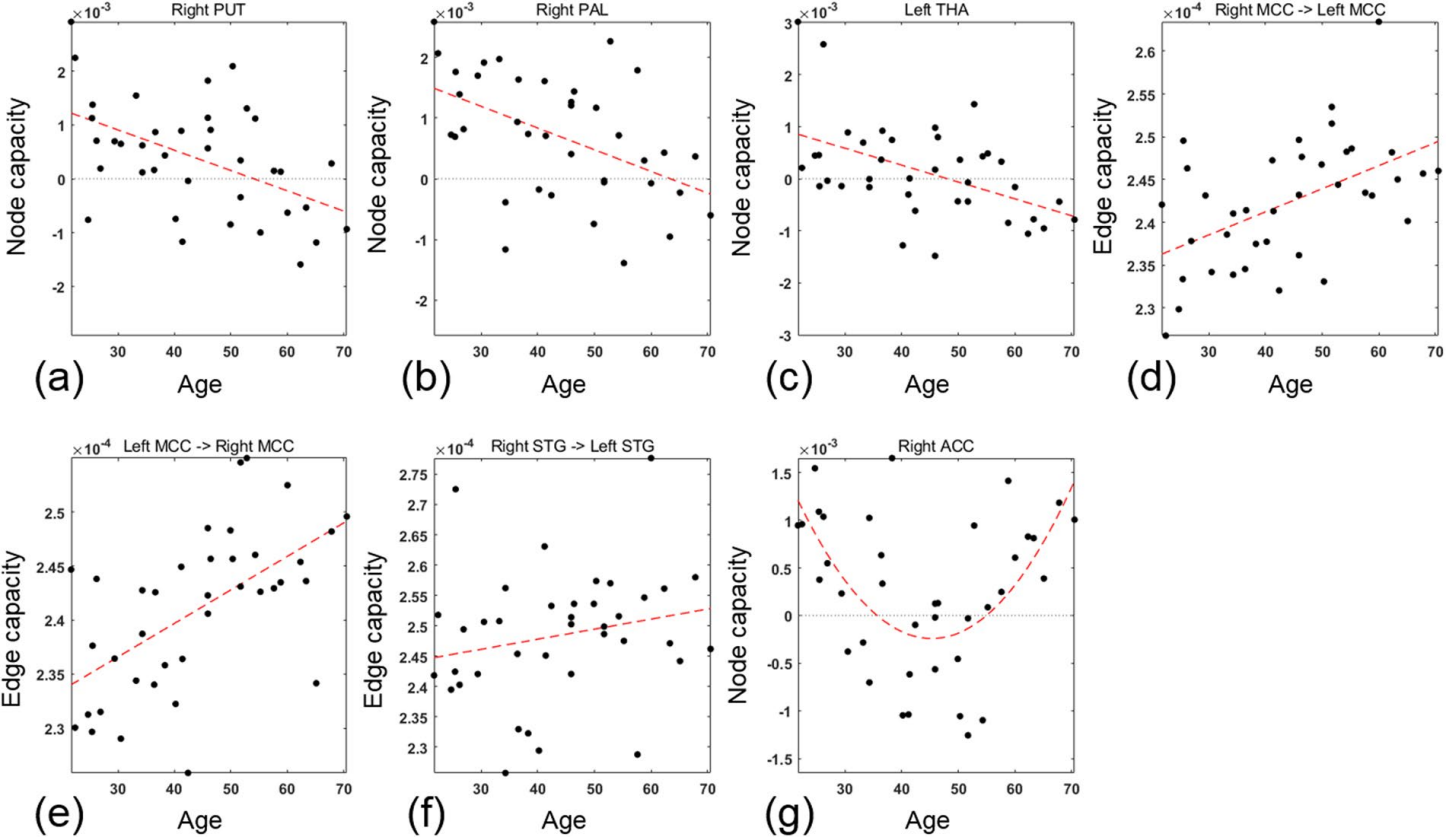
(**a**–**c**) Node capacities of right PUT and right PAL, and left THA in 38 resting state functional networks with respect to age. The node capacities of right PUT and PAL, and left THA significantly decreased with age (*p* < 0.05, FDR-corrected). (**d**–**f**) Edge capacities of edges directed from right MCC to left MCC, in the opposite direction, and from left STG to right STG with respect to age. These edge capacities increased with age (*p* < 0.05, FDR-corrected). (**g**) Node capacity of right ACC. The node capacity of right ACC was a U-shaped curve with respect to age (*p* < 0.05, FDR-corrected).

Edge and node capacities were correlated with age and nodes of significant correlation was shown in Fig. 5. The negative correlation with the age was found in the edges directed from the most of the brain regions to right PUT and pallidum (PAL), and left THA (*p* < 0.05, FDR-corrected). Correspondingly, the node capacity of right PUT and PAL, and left THA decreased with age as shown in Fig. 5(a–c) (*p* < 0.05, FDR-corrected). The edge capacities of edges directed to the left PUT and PAL, and right THA and the node capacities of the left PUT and PAL, and right THA also tended to be negatively correlated with age (*p* < 0.05, uncorrected). The positive correlation with age were found in the bidirectional edges between the left and right median cingulate cortex (MCC) and the edge from right superior temporal gyrus (STG) to left STG as shown in Fig. 5(d–f) (*p* < 0.05, FDR-corrected). Beyond this linear relation, as a trial, we also estimated a quadratic relationship between the edge capacity and the age. The capacities of most of edges directed to the right anterior cingulate cortex (ACC) had a U-shaped curve with respect to age (*p* < 0.05, FDR-corrected). It decreased to around 45 years of age and increased at older age. Correspondingly, the node capacity of the right ACC also had a U-shaped curve with respect to age as shown in Fig. 5(g). The minimum node capacity of the right ACC was also found at around 45 years of age.

### Simulations

We compared the performance of the volume entropy in comparison to that of the well-known global graph measures in distinguishing artificial networks with different topologies and different geometries. The global graph measures we compared here were global efficiency (*e*_*glo*_), average local efficiency (*e*_*loc*_), modularity (*Q*), functional entropy (*h*_*fun*_), spectral entropy (*h*_*spe*_), and volume entropy (*h*_*vol*_)^10,15,16^.

**Figure 6:**
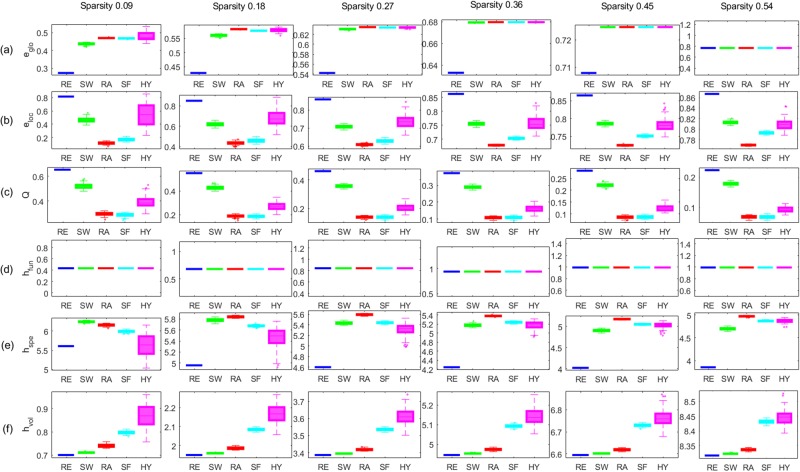
Comparison of graph measures using various artificial unweighted networks. Each panel shows the box plot of (**a**) global efficiency (*e*_*glo*_), (**b**) average local efficiency (*e*_*loc*_), (**c**) modularity (*Q*), (**d**) functional entropy (*h*_*fun*_), (**e**) spectral entropy (*h*_*spe*_), and (**f**) volume entropy (*h*_*vol*_). The sparsity of the unweighted network was 0.09, 0.18, 0.27, 0.36, 0.45, and 0.54 from left to right. The color of the line represents RE (blue), SW (green), RA (red), SF (cyan), and magenta (HY). The order of the five types of different unweighted networks was consistent for the sparsity when the graph property was measured by the modularity *Q* in (**c**) and the volume entropy *h*_*vol*_ in (**f**). However, the order was changed more than three times depending on sparsity in the other graph measures, *e*_*glo*_ in (**a**), *e*_*loc*_ in (b), and *h*_*spe*_ in (**e**). *h*_*fun*_ in (**d**) was exactly the same for all graph types at the fixed sparsity.

First, we generated artificial unweighted networks with different topologies such as a regular graph (RE), small-world graph (SW), random graph (RA), scale-free graph (SF), and hyperbolic graph (HY)^18^. Before estimating graph measures, we normalized edge distances to have the sum of edge distances 2. Then, we compared the performance of the six global graph measures in discriminating the five networks by Wilcoxon rank sum test. More details are in the Sec. 3 of the Supplementary Material. The results in Fig. 6 showed that the volume entropy *h*_*vol*_ and the modularity *Q* distinguished the five artificial unweighted networks consistently along the variety of the sparsity of the networks (*p* < 0.001, FDR-corrected), while the performance of the other global graph measures, *e*_*glo*_, *e*_*loc*_, *h*_*spe*_ and *h*_*fun*_ varied unpredictably depending on the sparsity. The global and local efficiencies, *e*_*glo*_ and *e*_*loc*_ distinguished RE, SW, and RA along the variety of the sparsity, but, they could not discriminate SF and HY from RA or others.

**Figure 7:**
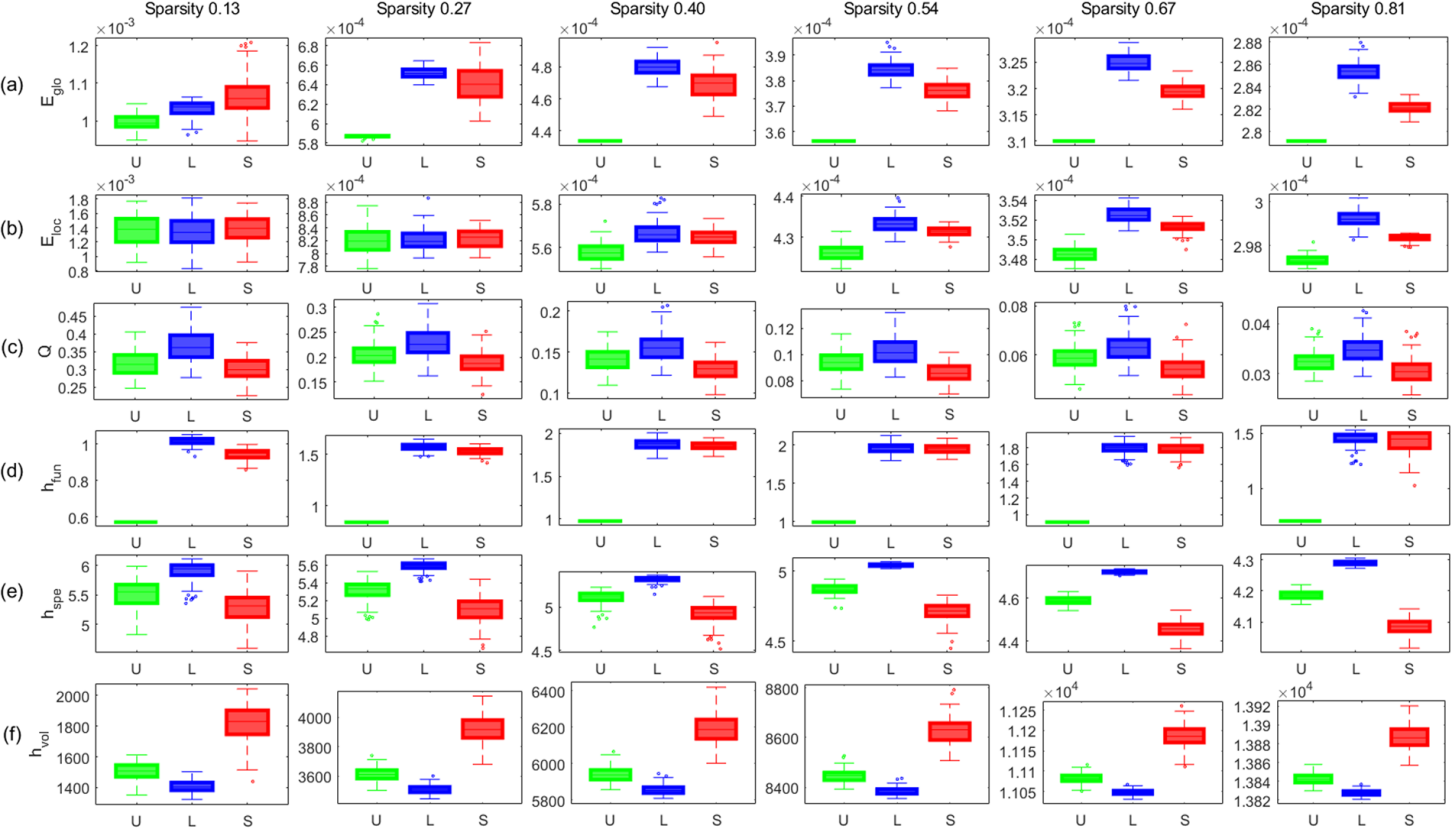
Comparison of graph measures using various artificial weighted networks. Each panel shows the box plot of (**a**) *e*_*glo*_, (**b**) *e*_*loc*_, (**c**) *Q*, (**d**) *h*_*fun*_, (**e**) *h*_*spe*_, and (**f**) *h*_*vol*_. The sparsity of the weighted network was 0.13, 0.27, 0.40, 0.54, 0.67, and 0.81 from left to right. The color of a line represents U (green), L (blue), and S (red). When the graph property was measured by *Q* in (**c**), *h*_*spe*_ in (**e**), and *h*_*vol*_ in (**f**), the order of the three types of weighted networks was consistent for the sparsity. However, the order was changed more than twice depending on sparsity in the other graph measures, *e*_*glo*_ in (**a**), *e*_*loc*_ in (**b**), and *h*_*fun*_ in (**d**).

Second, we compared the performance of the six graph measures in discriminating three types of artificial weighted networks with different geometries of hyperbolic networks. We generated an unweighted hyperbolic network, and set the edge weights in three different ways as follows. The first type of a network had uniform edge distance, denoted by U, the second type of a network had the edge distance that was proportional to the degree of the initial and terminal nodes, denoted by L, and the last type of a network had the edge distance that was inversely proportional to the degree, denoted by S. More details are in the Sec. 4 of the Supplementary Material. Thus, we can say that three networks had the same topology, but different geometries. The edge connecting with higher degree was longer in L, but shorter in S. Thus, we could assume that the information propagation was the fastest in S, followed by U and L. We normalized edge distances so that the sum of edge distances is equal to 2, and estimated graph measures. The results in Fig. 7 showed that the modularity *Q*, the spectral entropy *h*_*spe*_, and the volume entropy *h*_*vol*_ distinguished U, L, and S consistently along the variety of sparsity (*p* < 0.001, FDR-corrected). Especially, they distinguished them in the order, L, U, and S as we expected. However, only the volume entropy *h*_*vol*_ yielded the smaller value in L and larger value in S. Fastest information flow in S was represented by larger value of the volume entropy *h*_*vol*_ in S. The modularity *Q* showed the reverse order of values which made sense but showed much overlap, and the spectral entropy *h*_*spe*_ yielded the results in a reverse order compared with *h*_*vol*_, which was difficult to interpret. *e*_*glo*_, *e*_*loc*_, and *h*_*fun*_ also appeared to distinguish three weighted networks, however, the sparsity affected the order of the weighted networks in the results of *e*_*glo*_, *e*_*loc*_, and *h*_*fun*_.

## Discussion

### Relationship between volume entropy and complex graph measures

Firstly, the volume entropy was large when there were many edges, i.e., larger sparsity in a network. Simulation study of binary and weighted networks (Figs 6 and 7) showed that the varying sparsity affected the volume entropy much more (for example, from 1 to 7 *h*_*vol*_) than the network topology or geometry with the same sparsity. The sparsity also affected the other graph measures of global and local efficiencies and spectral and functional entropies but with much less variations (for example, from 0.5 to 1 in case of *e*_*glo*_). In our simulation study and later on in human brain studies, we used fully connected weighted networks, and minimized the effect of sparsity on the parameters including volume entropy *h*_*vol*_.

Secondly, the volume entropy was roughly proportional to the global efficiency, and inversely proportional to the local efficiency when the sparsity was low. When the volume entropy was applied to the binary networks with distinct topology, the order of graphs was RE < SW < RA for all the ranges of sparsities. The results of the simulations also showed that the global efficiency *e*_*glo*_ was the smallest in RE, followed by SW and RA, while the local efficiency *e*_*loc*_ was the opposite. The global efficiency was proportional to the characteristic path length, while the local efficiency was inversely proportional to the clustering coefficient^10^. According to the Watts-Strogatz model of the small world, the characteristic path length and the average clustering coefficient were the smallest in RE, followed by SW and RA^19^. *e*_*glo*_ could not characterize SF or HY networks and the values of *e*_*loc*_ for SF or HY networks could not be understood. If a network had high average clustering coefficient, but short characteristic path length as in SW, the information would not be propagated rapidly throughout the network as the information would whirl around only in the several nodes with strong clustering coefficients. That might have been the reason why the volume entropy *h*_*vol*_ of SW was smaller than those of RA (and also SF or HY).

Thirdly, the volume entropy *h*_*vol*_ was large when a network had hubs that allow all nodes in the network to be reachable within a few hops. Scale-free network (SF) and hyperbolic network (HY) known to have hubs had larger volume entropy than RE, SW, and RA on our simulation results (Fig. 6). SF and HY were networks with hubs which are contributing much to the exponential growth of the path of a network through which information were easily and rapidly delivered^20,21^.

Finally, the volume entropy *h*_*vol*_ of HY was even larger than that of SF. This was comprehensible as HY was known as a network with nodes having high clustering coefficients and heterogeneous degree distribution, while SF had only heterogeneous degree distribution^21^. In HY, there were many paths between highly clustered nodes. If the paths outgoing from these clustered nodes were properly created, the high local efficiency in HY would have contributed to fast information propagation, which was represented in larger *h*_*vol*_ of HY. Nodes with high clustering coefficients not only hold the information to themselves, but also create many paths in the graph within themselves. Paths out of these highly clustered nodes in HY will propagate information within brain networks, and of course, in this occasion *h*_*vol*_ will be higher. The volume entropy *h*_*vol*_ can be considered to represent (efficient) information flow all over the brain graph and also measure the efficiency of the hyperbolic brain graphs.

### Normalization of graph volume

Calculated volume entropy highly depends upon the number of edges and the sparsity and thus also the volume of the brain graph. In the results, we used the normalized volume entropy and it decreased significantly along with age. The volume of functional networks decreased with age (*p* < 0.05). If not normalized to the graph volume, the aging effect of volume entropy would have been confounded by the changes of graph volume which showed age-related decline too. However, interestingly unnormalized volume entropy did not show age-related decline changes (see in the Sec. 5 of the Supplementary Material). Thus, while the decline of brain graph volume with age meant that the average connection between brain regions became shorter, but after removing this confounding effect, the (normalized) volume entropy showed age-related decline meaning that the inherent topological structure of the brain network became increasingly inefficient with aging. Or this might also be interpreted that with aging, the connections came to be shorter (stronger) between brain regions, i.e., increased correlations between brain regions in order to compensate the age-related aggravation of inefficiency in topological composition of brain networks. Interestingly, we adopted graph volume and the normalization of volume entropy with graph volume $vol(N)=l(E)={\sum }_{e\in E}\,l(e)$, but the other parameters of complex graphs could have been normalized by graph volumes. We normalized the graph volumes when we calculated *e*_*glo*_, *e*_*loc*_, *Q*, *h*_*spe*_ and *h*_*fun*_ as well as our parameter of *h*_*vol*_.

### Comparison with the previous studies

Previous studies on resting-state functional connectivity have shown inconsistent results of global and local efficiencies across the lifespan^12,22–24^. In contrast, there were consistent reports of the age-related reorganization in the modular structure of a functional connectivity^22,25,26^. Especially, these previous studies found that the modularity decreased after 40 years of age^22,25,26^. In this study, the global and local efficiency, *e*_*glo*_, *e*_*loc*_, did not have relationships to the age both in the normalized metabolic and functional networks (see the Sec. 5 in the Supplementary Material). However, they were proportional to the age in the unnormalized functional networks (*p* < 0.05). This might be related with the fact that the volume of functional networks decreased with the age. Our result also showed the age-related decrease in the modularity of functional networks, but not in that of metabolic networks (*p* < 0.05).

The age-related change of modularity was not affected by the normalization by the volume of the network. The human brains have modular architecture^27–29^. Nodes within a module are densely connected, and the shortest path lengths between any nodes within a module are short. In contrast, nodes between modules are loosely connected, and the shortest path lengths between nodes in different modules are long. Since the clustering coefficients and the characteristic path lengths are estimated as average values for brain graphs, it would not be appropriate to use these parameters to represent the heterogeneous distribution of shortest path lengths in the modular graphs. However, the volume entropy was calculated by the fastest growth rate of the number of network paths in their universal covers of the graphs, and thus was not affected by such heterogeneity of brain networks. Volume entropy is proposed to be an appropriate network measure when comparing normalized networks with heterogeneous topological properties in their performances of information propagation.

### Edge and node capacities on a metabolic network

The sum of edge capacities in a brain network was one because it was the normalized eigenvector of the GMS of brain networks. Therefore, the increase or decrease of the edge capacity with age should be interpreted as the change of the relative proportion of the edge capacity in the whole brain, not the change of its absolute value. We assumed that the information flowed through the paths in the brain graph, and the amount of information going through the edge was positively related to the number of paths upon the edge.

The analysis on the metabolic network revealed that the role of the left ANG was significantly changed in the information propagation with aging. The left ANG showed negative node capacity in moderate degree in Y group, came to show positive node capacity in O group and the difference was significant between Y and O groups. The result meant that more information would flow into the ANG, which was known as the functional hub of default mode network (DMN), with aging^30^. With the limited number of subjects in this study and grouping these subjects to arbitrary two groups, Y and O, to find the aging effect and the use of strict criteria of statistical significance yielded only one node, the left ANG, to have the significant change between Y and O groups. Or this might be related with the report that the left hemisphere had less age-related decline than the right hemisphere^31^. In any case, this finding should be recapitulated further with the following studies as this was the first observation that a specific region is found to change its role along aging in its role of information source node to absorbing node using our GMS model of capacity estimations for edges and nodes.

### Edge and node capacities on a functional network

The volume entropy of the functional networks decreased with age. Thus, the functional network could have nodes and edges where the information propagation slowed down and became inefficient along with aging. Using GMS modeling and estimation of edge and node capacities of the brain nodes, we could find out the nodes or edges which contributed to these changes. Right PUT, right PAL and also left THA showed significant changes along aging from absorbing nodes (incoming dominant) to source nodes (outgoing dominant). Left PUT, left PAL and right THA tended to change along aging. The bilateral PUT, PAL, and THA received more information from the other nodes than that they sent between 20 s and the early 40 s and the information they sent to other nodes increased from the late 40 s to the early 60 s. We suggest that these changes of node capacity are associated with the decrease of volume entropy with aging. The circuit linking the PUT, PAL, THA, and cortical areas play a key role in motor ability across the human lifespan^32,33^. During normal aging, the basal ganglia-thalamocortical circuits change structurally and/or functionally as well as in Alzheimer’s and Parkinson’s diseases^33–36^. We speculate that the changes of information flow, globally and in basal ganglia/thalamus would be indicative of these changes in normal aging.

The ACC is one of the key areas involved in cognitive and emotional processing^37,38^. On a resting-state fMRI, functional connectivity between the ACC and default mode network decreased in association with the deficit of cognitive processing in aging, while functional connectivity increased between the ACC and the emotion-related brain regions such as the STG, inferior frontal gyrus (IFG), PUT, and amygdala (AMYG) in association with the well-maintained emotional well-being in aging^38^. In our analysis to find the role of node in aging using quadratic model, right ACC was revealed to show decrease of it node capacity until around 45 years of age, but increase along aging after 45 years of age (Fig. 5(g)). The right ACC was initially absorbing node but sent more information to the other nodes than it received from the late 30 s to the mid-50s. In older ages, the right ACC received more information than it sent. We propose that our analysis results would unravel the roles and the changes of nodes as we yielded directed brain graphs.

The information capacities of bidirectional edges between the right and left MCCs had a linear relationship with age. The older the subject was, the more likely the bilateral MCCs would receive more information from the other brain regions (*p* < 0.1, uncorrected). The results meant that the amount of the information from the contralateral MCC was significant among all received information in the MCC. The MCC is related to environmental monitoring and response selection^39,40^. We speculate that age-related changes of edge capacities are related with the age-related change in social decision-making of humans^41^. The edge capacity from the left to the right STGs also increased with age. In the brain networks on resting fMRI, the node capacity of only the right STG tended to increase with age (*p* < 0.05, uncorrected), while that of left STG did not. The older the subject was, the more the right STG tended to receive information from the other nodes, especially from the left STG. The STG is involved in language processing, multisensory integration, and social perception^42,43^. The dysfunction of the right STG was found to be related with the social cognition deficit in normal aging^44^.

## Conclusions

In this study, we introduced a new network measure, called a volume entropy. It measured the fastest growth rate of paths in a network through which the information was propagated over a brain. The larger the volume entropy was, the more information was propagated in a specified graph. Thus, it could be regarded as a new graph measure of efficiency of information flow/propagation within a specific volume of brain graph. The simulation study results showed that the volume entropy was an appropriate parameter to measure the efficiency of networks with heterogeneous degree and clustering of nodes such as scale-free and hyperbolic graphs. The information flow in a graph was modelled by the GMS associated with a newly defined edge-transition matrix. The volume entropy was estimated by the stationary equation of this GMS. At the same time, we could obtain the stationary distribution of information flow in a network. It provided a new insight of how much and in what direction the information flowed on edges and nodes of the brain network. We named these numerical outputs as edge capacity and node capacity. Node capacity was defined simply as the difference of the sum of incoming edge capacities and the sum of outgoing edge capacities of a specified node.

The edge capacity made the directed weighted graph induced by the stationary distribution of the GMS and were found to be highly depended on the terminal node of an edge. The node capacity can visualize these directed graphs, in which we put the blue color for inward dominance as absorbing nodes and red color for outward dominance as source nodes and the size of the disc represented the size of node capacity. We speculated that if we mathematically delineate the relationship between the edge capacity and the well-known complex network measures, the biological meaning of edge capacity might be able to be understood better. Instead we did simulation studies and found that the brain graphs should be normalized to yield volume entropy (as global measure of information flow or propagation) and edge or node capacities. Especially, if we are going to compare the volume entropy (or edge or node capacities) of different individuals or along aging or between groups, we emphasize the needs of normalizing brain graph volumes. This was the simplest but important result of this investigation and was derived from the theoretical definition of volume entropy. As was expected, the volume entropy of various types of artificial networks including small world, scale-free or hyperbolic structures, showed increasing values along the varying types of complex networks, while other complex network measures did not.

The significance in the differences of edge capacities or node capacities between the groups Y and O in metabolic networks or along aging in functional networks was rarely found due to the small number of subjects. However, we could find that the volume entropy tended to decrease with aging both in the metabolic and functional networks. Contributing edges and nodes to affect this decrease were of utmost interest, but the nodes and edges contributing to the aging information flow of the functional and metabolic networks were different. We interpreted the functional and metabolic networks separately though they were from the same individuals acquired from the hybrid PET/MRI machines. And as expected, PET metabolic data were analyzed as groups Y and O, and resting fMRI were analyzed for all the individuals individually. The comparison to find the relationship of PET and resting fMRI are warranted. We recognize that resting fMRI and PET, though acquired at the same scan period, represent different time period (40 to 60 minutes before scanning for PET after FDG injection) and point (3 minutes during scanning in the gantry for resting fMRI).

The proposed method can be applied to the brain imaging data of various disease groups as well as that of normal controls. We expect that we will be able to unravel which edges or which nodes are the culprit for the abnormality of information exchange in the brains of the many diseases in which no topographical or connectivity abnormality had been disclosed yet, as our new method will produce directed weighted graph and the visualized graph representation of nodes. We propose that this new method reveal the information flow of an effective functional connectivity of which connections represent the causal relationship between brain regions, which is under active progress now.

## Methods

### Resting state fMRI and PET data sets

PET and fMRI data were simultaneously acquired from 38 healthy normal subjects (M/F: 19/18, mean age: 43.9 ± 13.9, age range: 22–71) using a Siemens Biograph mMR 3T scanner (Siemens Healthcare Sector, Germany). All experiments were performed in accordance with ethical guidelines and regulations that have their origin from the Declaration of Helsinki. This study was approved by the Institutional Review Board (IRB) at Seoul National University Hospital (IRB No. 1210-011-431). All the participants completed the written informed consent before the experiment and received monetary compensation for their participation.

MR images had 116 volume of images per a subject. The first 4 volumes were discarded among 116 volumes and 112 volume of images a subject were used for network analysis. After preprocessing using the AFNI^45^ and the FSL^46^, we parcellated the brain into 116 regions of interest (ROIs) according to automated anatomical labelling (AAL)^47^. Among the 116 regions, 90 brain regions were selected as the nodes and 26 cerebellar regions were not included in a graph (the number of nodes, *p* = 90). The measurement of each node was obtained by averaging blood-oxygen-level dependent (BOLD) signals in the ROI of fMRI data. Each node had *n* measurements, which were the number of time points a subject in fMRI data (*n* = 112). The measurement vectors of 90 ROIs were written by ${{\boldsymbol{x}}}_{1}^{j},\,\ldots \,\,{{\boldsymbol{x}}}_{p}^{j}\in {{\mathbb{R}}}^{n}$ of the *j*th subject (*j* = 1, …, 38, *p* = 90, *n* = 112).

PET images were preprocessed using the Statistical Parametric Mapping (SPM8) for image registration and PVElab software for partial volume correction^48^. The image intensity of gray matter was globally normalized to 50. The measurement of a node was obtained by averaging FDG uptakes in the corresponding ROI. We divided the data into two groups, young (age: 32.2 ± 6.9) and old (age: 55.6 ± 7.7) depending on whether a subject was over age 45. The number of subjects in each group was 19. We had the measurement vectors of PET data, $\,{{\boldsymbol{x}}}_{1}^{Y},\,\ldots ,\,{{\boldsymbol{x}}}_{p}^{Y}\in {{\mathbb{R}}}^{n}$ for young group, denoted by Y, and $\,{{\boldsymbol{x}}}_{1}^{O},\,\ldots ,\,{{\boldsymbol{x}}}_{p}^{O}\in {{\mathbb{R}}}^{n}$ for old group, denoted by O (*p* = 90, *n* = 19).

### Distance of brain network

The edge weight between two nodes *i* and *t* is estimated by the Gaussian kernel based on Pearson correlation:

3 $${w}_{it}=k({{\boldsymbol{x}}}_{i},\,{{\boldsymbol{x}}}_{t})=\exp (-\frac{1-corr({{\boldsymbol{x}}}_{i},{{\boldsymbol{x}}}_{t})}{{\sigma }_{i}{\sigma }_{t}}),$$

where *corr*(***x***_*i*_, ***x***_*t*_) is the Pearson correlation between two measurement vectors ***x***_*i*_ and ***x***_*t*_ and *σ*_*i*_ is the width of Gaussian kernel. Because $1-corr({{\boldsymbol{x}}}_{i},\,{{\boldsymbol{x}}}_{t})=\frac{1}{2}{\Vert \frac{{{\boldsymbol{x}}}_{i}}{\Vert {{\boldsymbol{x}}}_{i}\Vert }-\frac{{{\boldsymbol{x}}}_{t}}{\Vert {{\boldsymbol{x}}}_{t}\Vert }\Vert }^{2}$, $\sqrt{1-corr({{\boldsymbol{x}}}_{t},\,{{\boldsymbol{x}}}_{t})}$ is conditionally negative semi-definite for $\,{{\boldsymbol{x}}}_{i},\,{{\boldsymbol{x}}}_{t}\in {{\mathbb{R}}}^{n}$ ^49^. The Gaussian kernel based on correlation in (3) is positive definite for all *σ*_*i*_ > 0 and satisfies Mercer’s theorem^49^. Thus, it transforms the original data in a nonlinear manifold into a higher dimensional feature space where the transformed features have a linear representation. The distance of the kernel *w*_*it*_ is estimated by a kernel trick^50^:

4 $$\begin{array}{rcl}{d}_{it} & = & \Vert \varphi ({{\boldsymbol{x}}}_{i})-\varphi ({{\boldsymbol{x}}}_{t})\Vert \\  & = & {\langle \varphi ({{\boldsymbol{x}}}_{i})-\varphi ({{\boldsymbol{x}}}_{t}),\varphi ({{\boldsymbol{x}}}_{i})-\varphi ({{\boldsymbol{x}}}_{t})\rangle }^{\mathrm{1/2}}\\  & = & {[k({{\boldsymbol{x}}}_{i},{{\boldsymbol{x}}}_{i})+k({{\boldsymbol{x}}}_{t},{{\boldsymbol{x}}}_{t})-2k({{\boldsymbol{x}}}_{i},{{\boldsymbol{x}}}_{t})]}^{\mathrm{1/2}}\\  & = & \sqrt{2-2k({{\boldsymbol{x}}}_{i},\,{{\boldsymbol{x}}}_{t})}\mathrm{.}\end{array}$$

If an edge *e* connects two nodes *i* and *t*, *d*_*it*_ is also denoted by *l*(*e*).

The kernel-based distance is a Euclidean distance between two nodes in a higher dimensional feature space. When the kernel width is small in (3), the local neighbors that are highly positively correlated in the original data space are more clearly separated in the feature space, while non-local neighbors are not. The kernel width *σ*_*i*_ in (3) is determined by the tenth smallest one among all 1 − *corr*(***x***_*i*_, ***x***_*t*_) (*t* = 1, …, *i* − 1, *i* + 1, *p*)^51^.

The 38 brain graphs of 38 subjects were constructed from fMRI data by the kernel-based distance in (4) and (3). Two brain graphs of two groups, Y and O were constructed from PET data. We call the brain graphs constructed by fMRI and PET data functional and metabolic graphs, respectively.

### Volume entropy

Suppose that ${\mathscr{N}}={\mathscr{N}}(V,\,E,\,l)$ is a connected finite graph with the node set *V*, the edge set *E*, and the edge distance function *l*. We assume that a graph ${\mathscr{N}}$ does not have any terminal node. In a graph ${\mathscr{N}}$, each edge *e* ∈ *E* has an associated value, called the distance of the edge *l*(*e*). Let *S* be a subset of edges with multiplicities, where each edge can be counted several times. The volume of *S* is defined by $l(S)={\sum }_{e\in S}\,l(e)$. For example, *S* = {*e*, *e*, *f*} for *e*, *f* ∈ *E* is allowed and the volume of *S* is 2*l*(*e*) + *l*(*f*). Edge *e* is assumed to have an orientation from the initial node *i*(*e*) ∈ *V* to the terminal node *t*(*e*) ∈ *V*. For a given oriented edge *e* from *i*(*e*) to *t*(*e*), we denote by $\bar{e}$ the oriented edge from $i(\bar{e})=t(e)$ to $t(\bar{e})=i(e)$. Note that for any *e* ∈ *E*, both *e* and $\bar{e}$ exist in ${\mathscr{N}}$. We normalize the volume of *E* by *l*(*E*) = 2.

The sequence of *n* consecutive edges without backtracking is denoted by a path ${\mathscr{P}}={e}_{1}{e}_{2}\cdots {e}_{n}$ (${e}_{j+1}\ne \overline{{e}_{j}}$, *e*_*j*_ ∈ *E*). The set of all possible paths of length *r* starting from a node *v*_0_ ∈ *V* in ${\mathscr{N}}$ has a structure of a tree, which we denote by *B*(*v*_0_, *r*). Because ${\mathscr{N}}$ is assumed to have no terminal node, the number of possible paths *B*(*v*_0_, *r*) increases exponentially as *r*→∞. The limit of the ball *B*(*v*_0_, *r*) as *r* → ∞ is called the universal covering tree of ${\mathscr{N}}$.

The volume entropy *h*_*vol*_ is defined as^14^

5 $${h}_{vol}=\mathop{\mathrm{lim}}\limits_{r\to \infty }\frac{\mathrm{log}\,l(B({v}_{0},\,r))}{r}\mathrm{.}$$

The volume entropy *h*_*vol*_ does not depend on *v*_0_. When *h*_*vol*_ > 0, it is easy to see that *l*(*B*(*v*_0_, *r*)) is concentrated on the outer shell, i.e.

$$l(B({v}_{0},\,r)-B({v}_{0},\,r-{r}_{0}))\sim {e}^{{h}_{vol}r}-{e}^{{h}_{vol}(r-{r}_{0})}={e}^{{h}_{vol}r}\mathrm{(1}-{e}^{-{r}_{0}}\mathrm{).}$$

It follows that

$${h}_{vol}=\mathop{\mathrm{lim}}\limits_{r\to \infty }\frac{\mathrm{log}\,l(B({v}_{0},\,r)-B({v}_{0},\,r-{r}_{0}))}{r},\,{\rm{for}}\,{\rm{any}}\,{r}_{0} > 0.$$

Note also that

$${h}_{vol}=\mathop{\mathrm{lim}}\limits_{r\to \infty }\frac{\mathrm{log}\,{N}_{r}({v}_{0})}{r},$$

where *N*_*r*_(*v*_0_) is the number of paths of length *r* in the graph starting from *v*_0_, since

$${r}_{0}{N}_{r-{r}_{0}}({v}_{0})\le l(B({v}_{0},\,r)-B({v}_{0},\,r-{r}_{0}))\le {r}_{0}{N}_{r}({v}_{0}\mathrm{).}$$

In other words, the volume entropy *h*_*vol*_ is the exponential growth rate of the number of paths *N*_*r*_(*v*_0_) as *r* → ∞. The volume entropy is also represented by the stationary equation of the GMS in (2). The relationship between (2) and (5) was proved in Theorem 4 in Lim’s paper^14^.

### Toy example

**Figure 8:**
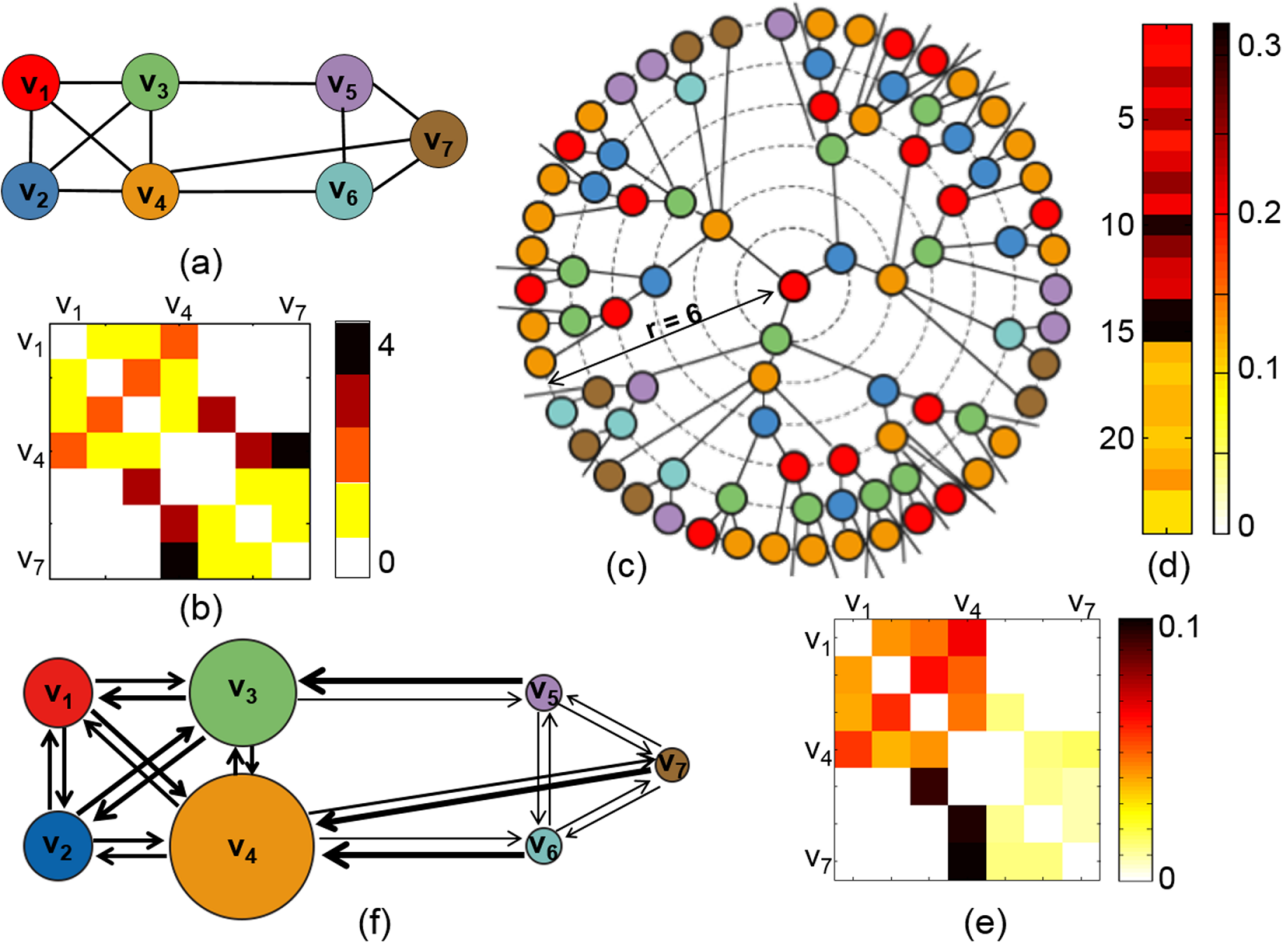
(**a**) Toy example of a weighted graph. (**b**) Distance matrix of (**a**). (**c**) Growth of network paths from *v*_1_ in a universal covering tree. (**d**) Eigenvector *z* of the edge-transition matrix of (**a**) in (1). (**e**) Edge capacity matrix calculated from *z* in (**d**). (**f**) Directed network that shows information flow on the weighted network (**a**). The size of a node is proportional to its node capacity, and the line width of an edge is proportional to its edge capacity.

Suppose that a weighted graph and its distance matrix are given in Fig. 8(a,b). We assume that the information flows through the edges on the network. If the information starts to flow from *v*_1_, the information will flow to *v*_2_, *v*_3_, and *v*_4_ along the connected edges in (a). After arriving at *v*_2_, *v*_3_, and *v*_4_, the information will flow to the next connected nodes. The path will lead to *v*_3_ and *v*_4_ at *v*_2_, and the path will lead to *v*_2_, *v*_4_, and *v*_5_ at *v*_3_. This growth of network paths started from *v*_1_ is shown in Fig. 8(c). If the time *r* goes to infinity, the resulting tree of network paths is called a universal covering tree. We take a ball of radius 6 of universal covering tree, denoted by *B*(*v*_0_ = *v*_1_, *r* = 6), and plot it in Fig. 8(c). If the time *r* goes to infinity, the number of network paths will increase exponentially. The exponential growth rate of the number of network paths is called a volume entropy.

The weighted graph in Fig. 8(a) had 7 nodes and 12 edges. To estimate a volume entropy in (2), we assume that each undirected edge consists of bidirectional edges with the same distance. Then, the number of oriented edges is *q* = 12 ⋅ 2 = 24. The edge-transition matrix ***L***(*h*_*vol*_) in (1) is a 24 × 24 dimensional sparse matrix, and its eigenvector *z* is a 24-dimensional vector as shown in Fig. 8(d). We normalize *z* by dividing each element in *z* by the sum of *z*, and obtain the edge capacity matrix as shown in Fig. 8(e). The directed network derived from the edge capacity matrix is shown in (f). In Fig. 8(f), the line width of an edge is proportional to its edge capacity in (e), and the size of a node is proportional to its node capacity.

In the given weighted graph in Fig. 8(a), the node sets {*v*_1_, *v*_2_, *v*_3_, *v*_4_} and {*v*_5_, *v*_6_, *v*_7_} form a module where all possible edges are connected, respectively called A and B for convenience. If we define the module size by the number of nodes in a module, the module size of A and B is 4 and 3, respectively. The size of A is larger than that of B. Thus, more paths will be generated in A than in B in the universal covering tree in (c). Moreover, the number of paths from B to A will be larger than that from A to B because the length of a path in B is shorter than that in A, and there are more paths out of B in the universal covering tree. Thus, the capacities of edges from *v*_5_ to *v*_3_, from *v*_7_ to *v*_4_, and from *v*_6_ to *v*_4_ are larger than that of the opposite directions. In the directed network of information flow in Fig. 8(f), the bidirectional edges within a module, A or B have similar line width (edge capacity), however, the line width of edges directed from A to B is much smaller than that from B to A.

### Normalization of graph volume

Suppose that a network $\tilde{{\mathscr{N}}}(V,\,E,\,\tilde{l})$ is given. The volume of a network is denoted by $vol(\tilde{{\mathscr{N}}})=\tilde{l}(E)={\sum }_{e\in E}\,\tilde{l}(e\mathrm{).}$ The network $\tilde{{\mathscr{N}}}(V,\,E,\,\tilde{l})$ is normalized to a network ${\mathscr{N}}(V,\,E,\,l)$ by reweighing edge distances by $l(e)=2\tilde{l}(e)/vol(\tilde{{\mathscr{N}}})$ for all *e* ∈ *E*. The normalized network ${\mathscr{N}}(V,\,E,\,l)$ has its volume $vol({\mathscr{N}})=2$. The stationary equation of GMS in the normalized network ${\mathscr{N}}$ is written in (2). We can rewrite the stationary equation in (2) by replacing *l*(*f*) with $\frac{2\tilde{l}(f)}{vol(\tilde{{\mathscr{N}}})}$ as follows:

6 $$\begin{array}{rcl}{z}_{e} & = & \sum _{f}\,{a}_{ef}{e}^{-{h}_{vol}\frac{2\tilde{l}(f)}{vol(\tilde{{\mathscr{N}}})}}{z}_{f}\\  & = & \sum _{f}\,{a}_{ef}{e}^{-\frac{2{h}_{vol}}{vol(\tilde{{\mathscr{N}}})}\tilde{l}(f)}{z}_{f}\\  & = & \sum _{f}\,{a}_{ef}{e}^{-{\tilde{h}}_{vol}\tilde{l}(f)}{z}_{f}\mathrm{.}\end{array}$$

Then, the volume entropy of the unnormalized network $\tilde{{\mathscr{N}}}$, denoted by ${\tilde{h}}_{vol}$, is proportional to the volume entropy of normalized network ${\mathscr{N}}$, *h*_*vol*_ as follows:

$${\tilde{h}}_{vol}=\frac{2}{vol(\tilde{{\mathscr{N}}})}{h}_{vol}\mathrm{.}$$

The stationary distribution *z* does not depend on the normalization of a network. In this study, we estimated the normalized volume entropy for all normalized networks.

## Electronic supplementary material

Supplementary material
