# Supplementary material for: Volume entropy for modeling information flow in a brain graph
Source: Sci Rep. 2019 Jan 22;9:256. doi: 10.1038/s41598-018-36339-7 (PMC6342973; doi:10.1038/s41598-018-36339-7)
Supplement: Supplementary file 1 — Supplementary material [file 41598_2018_36339_MOESM1_ESM.pdf]

Supplementary material of the manuscript, ‘Volume entropy for modeling information flow in a brain graph’

Hyekyoung Lee[1,2*], Eunkyung Kim[1,3], Seunggyun Ha[2], Hyejin Kang[2,4], Youngmin Huh[2,5], Youngjo Lee[4,6], Seonhee Lim[8*], and Dong Soo Lee[2,5,7,9*]

[1]Biomedical Research Institute, Seoul National University Hospital,
[2]Department of Nuclear Medicine, Seoul National University College of Medicine,
[3]Department of Rehabilitation Medicine, Seoul National University College of Medicine,
[4]Data Science for Knowledge Creation Research Center, Seoul National University,
[5]Interdisciplinary Program in Cognitive Science, Seoul National University,
[6]Department of Statistics, College of Natural Sciences, Seoul National University,
[7]Department of Molecular Medicine and Biopharmaceutical Sciences, Graduate School of Convergence Science and Technology, Seoul National University,
[8]Department of Mathematical Sciences, Seoul National University, Seoul
[9]Korea Brain Research Institute, Daegu, Republic of Korea
[*]hklee.brain@gmail.com, slim@snu.ac.kr, dsl@snu.ac.kr

1 Nodes

The information of nodes are summarized in Table 1 in the supplementary material. The location and color of nodes are shown in Fig. 1 in the supplementary material.

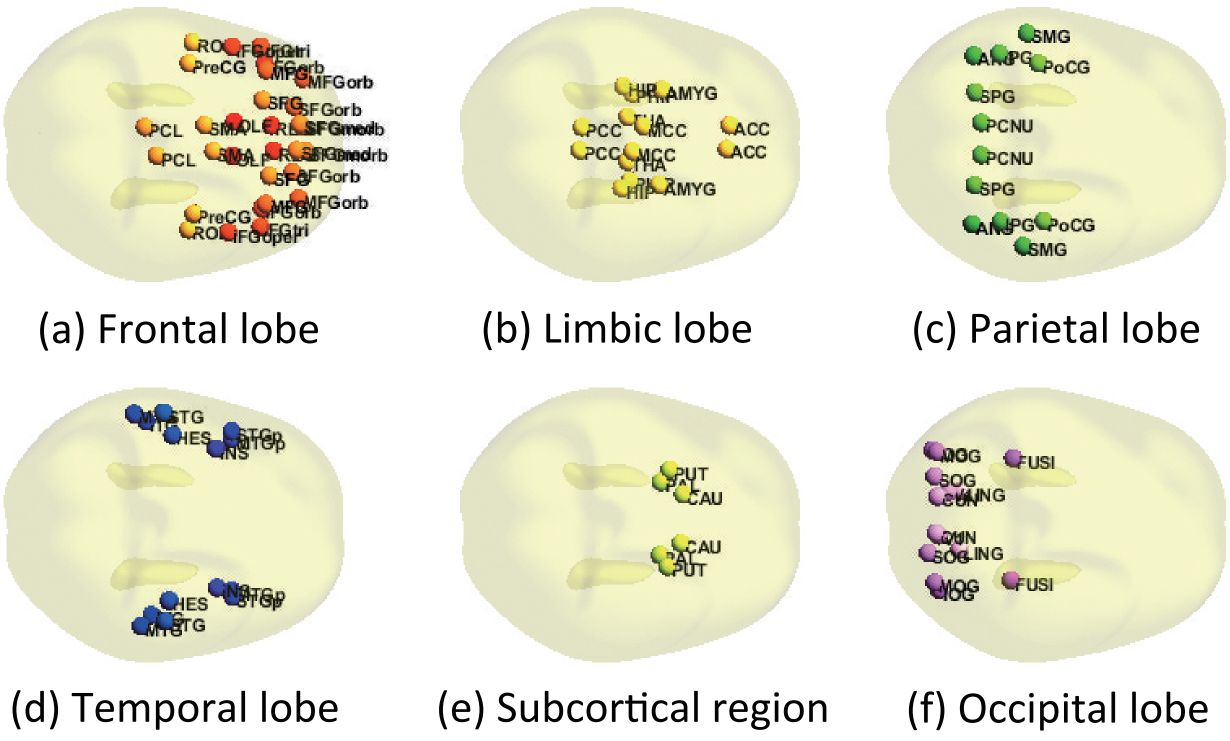

Figure 1. Color of node. (a) Frontal lobe (red), (b) limbic lobe (yellow), (c) parietal lobe (green), (d) temporal lobe (blue), (e) subcortical region (yellow-green), and (f) occipital lobe (purple).

Table 1. Information of 90 nodes

Index (R,L)	Lobe	abbrev.	Name
1, 90	F	OLF	Olfactory cortex
2, 89	F	REG	Gyrus rectus
3, 88	F	IFGorb	Inferior frontal gyrus, orbital part
4, 87	F	IFGoper	Inferior frontal gyrus, opercular part
5, 86	F	IFGtri	Inferior frontal gyrus, tri gular part
6, 85	F	MFGorb	Middle frontal gyrus, orbital part
7, 84	F	MFG	Middle frontal gyrus
8, 83	F	SFGorb	Superior frontal gyrus, orbital part
9, 82	F	SFGmorb	Superior frontal gyrus, medial orbital
10, 81	F	SFGmed	Superior frontal gyrus, medial
11, 80	F	SFG	Superior frontal gyrus, dorsolateral
12, 79	F	PCL	Paracentral lobule
13, 78	F	SMA	Supplementary motor area
14, 77	F	PreCG	Precentral gyrus
15, 76	F	ROL	Rolandic operculum
16, 75	L	ACC	Anterior cingulate and paracingulate gyri
17, 74	L	MCC	Median cingulate and paracingulate gyri
18, 73	P	PoCG	Postcentral gyrus
19, 72	P	SPG	Superior parietal gyrus
20, 71	P	PCNU	Precuneus
21, 70	P	IPG	Inferior parietal, but supramarginal and angular gyri
22, 69	P	SMG	Supramarginal gyrus
23, 68	P	ANG	Angular gyrus
24, 67	T	STG	Superior temporal gyrus
25, 66	T	HES	Heschl gyrus
26, 65	T	MTG	Middle temporal gyrus
27, 64	T	ITG	Inferior temporal gyrus
28, 63	T	STGp	Temporal pole: superior temporal gyrus
29, 62	T	MTGp	Temporal pole: middle temporal gyrus
30, 61	T	INS	Insula
31, 60	B	CAU	Caudate nucleus
32, 59	B	PUT	Lenticular nucleus, putamen
33, 58	B	PAL	Lenticular nucleus, pallidum
34, 57	L	THA	Thalamus
35, 56	L	AMYG	Amygdala
36, 55	L	HIP	Hippocampus
37, 54	L	PHIP	Parahippocampal gyrus
38, 53	L	PCC	Posterior cingulate gyrus
39, 52	O	FUSI	Fusiform gyrus
40, 51	O	IOG	Inferior occipital gyrus
41, 50	O	MOG	Middle occipital gyrus
42, 49	O	SOG	Superior occipital gyrus
43, 48	O	V1	Calcarine fissure and surrounding cortex
44, 47	O	CUN	Cuneus
45, 46	O	LING	Lingual gyrus

2 Edge and node capacities on 38 functional networks

The edge capacity and node capacity of 38 functional networks constructed by fMRI data were respectively shown in Fig. 2 and 3 in the supplementary material. The subjects were sorted in the ascending order of their age. The age of each subject was written above each figure. In Fig. 2, the minimum and maximum values of all edge capacity matrices are respectively 1.897×10^{-4} and 3.62×10^{-4} that are represented by dark red and light yellow. In Fig. 3, the size of nodes was proportional to the absolute of node capacity, and the color of nodes represented positive (blue) and negative (red) node capacities.

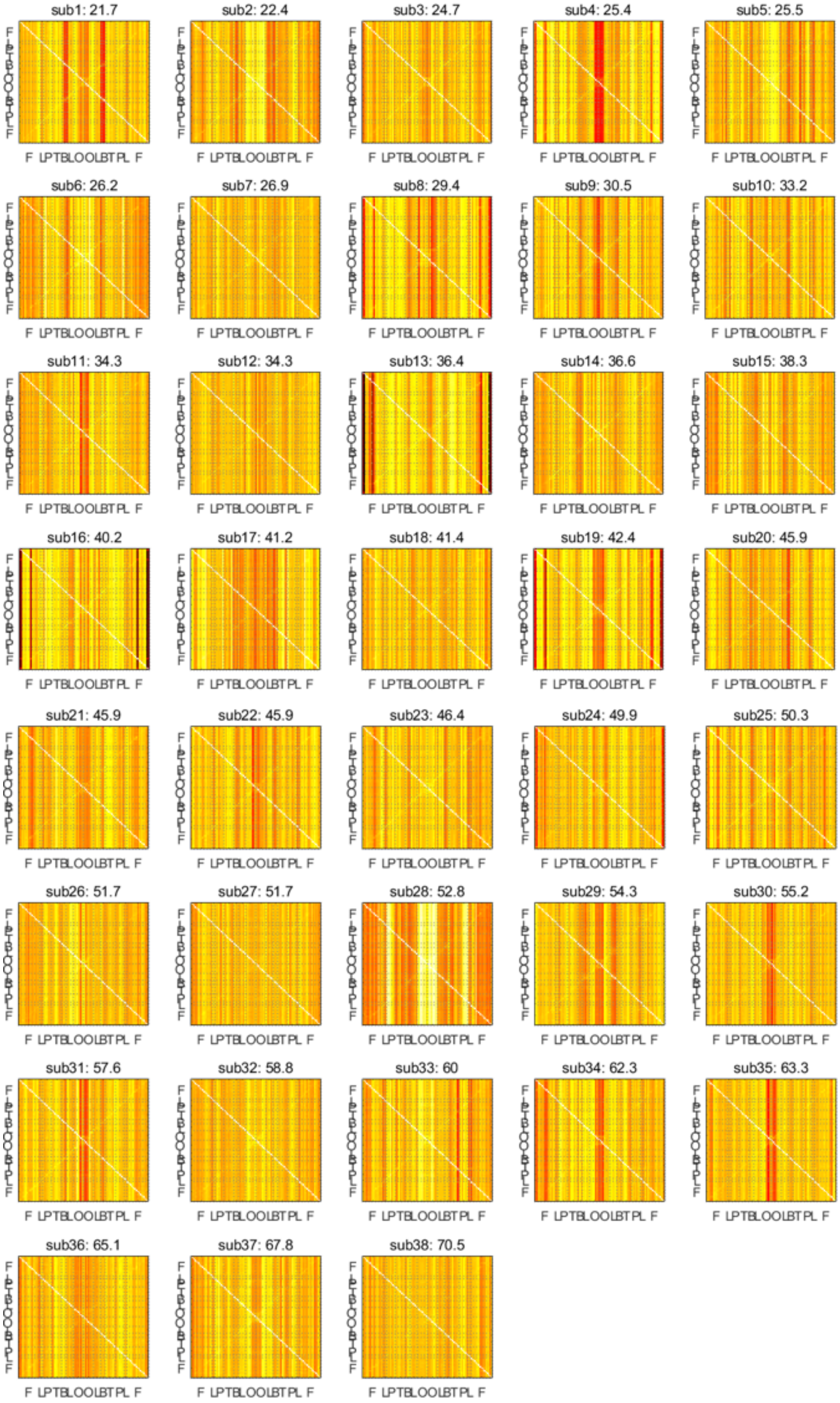

Figure 2. Edge capacity matrices of 38 functional networks. The range of edge capacities is between 1.897×10^{-4} and 3.62×10^{-4}. As the edge capacity increases, the color is changed from dark red to light yellow.

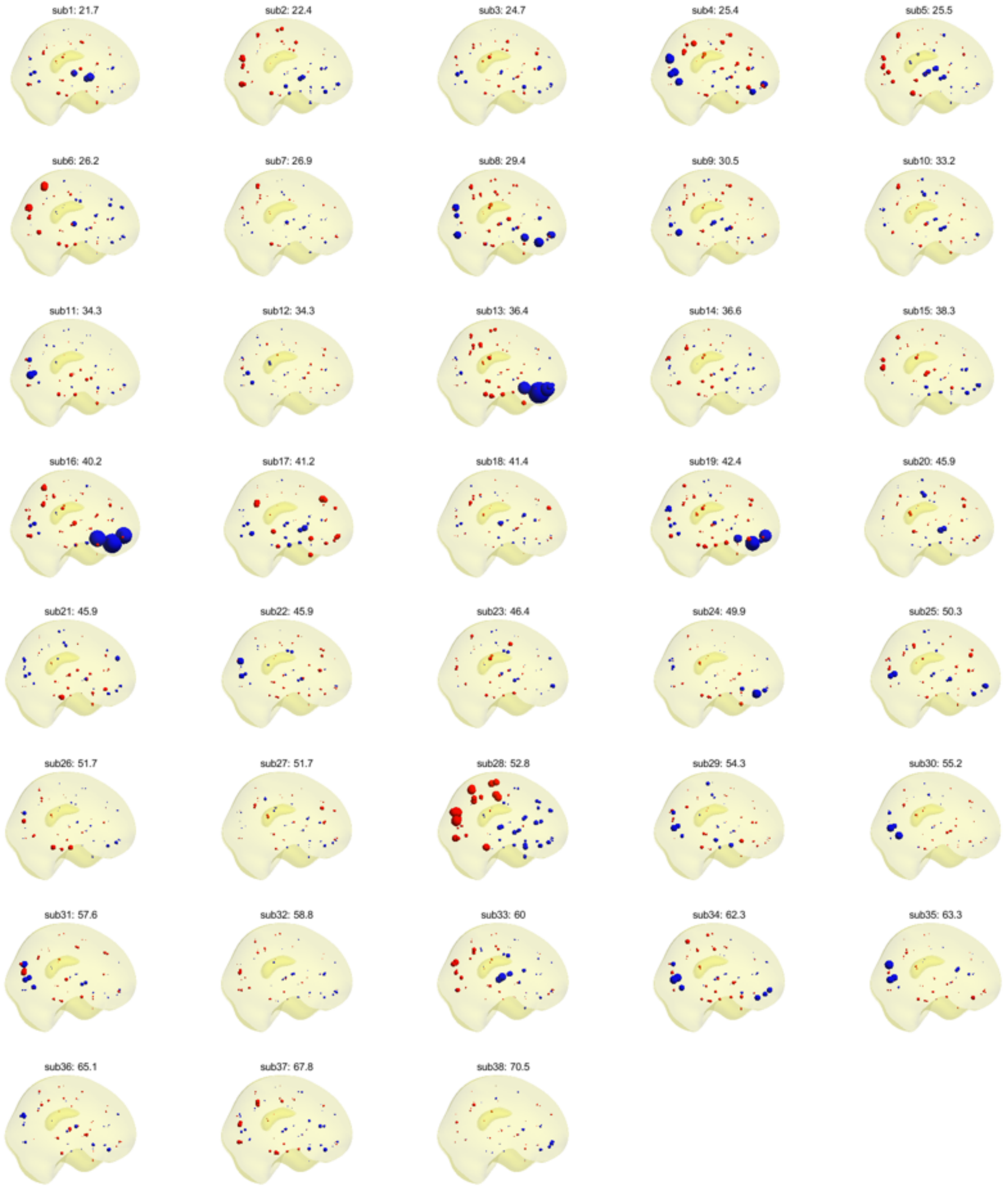

Figure 3. Node capacity of 38 functional networks. The network is sorted in the ascending order of the subject's age. The age of each subject was written above each figure. The size of nodes was proportional to the absolute of node capacity, and the blue and red nodes represented positive and negative node capacities, respectively.

3 Simulation using artificial binary networks

In this simulation, we compared the performance of six global graph measures in distinguishing five artificial unweighted graphs with varying the sparsity of a graph. The six different global graph measures were as follows:

- global efficiency e_{glo},
- average local efficiency e_{loc},
- modularity Q,
- functional entropy h_{fun}[1],
- spectral entropy h_{spe}[2], and
- volume entropy h_{vol} (the proposed method),

The five artificial unweighted graphs that were used for the comparison of performance were as follows:

- regular graph (RE),
- small-world graph (SW),
- random graph (RA),
- scale-free graph (SF), and
- hyperbolic graph (HY).

RE is an unweighted graph where all nodes have the same degree. SW is a globally and locally efficient graph with short characteristic path length and large average clustering coefficient[3]. SF has heterogeneous degree distribution with a few number of heavily linked nodes, termed hubs, but many nodes with few connections[4]. Hubs make a great contribution to propagating information quickly throughout a network. On the other hand, it is vulnerable to targeted attacks on hubs. Thus, SF is known to be globally efficient and locally inefficient. HY is known to have both strong heterogeneity and high clustering coefficient[5]. It can be thought as a maximally efficient unweighted graph. These unweighted graphs were generated by CNM matlab toolbox[6].

The number of nodes was fixed by $p = 90$. The sparsity, which was the ratio of the number of edges to the number of maximally possible edges, was varied from 0.04 to 0.90. All nodes in a graph should have more than three edges for the estimation of volume entropy[7]. If there were nodes with degree less than three in the generated graph, we randomly took an edge connecting nodes with degree more than four and rewired it to a node with degree less than three. In this way, we generated 150 artificial unweighted graphs for each sparsity and each graph type. After five measures were estimated in each graph, Wilcoxon rank sum test was performed to assess the statistical difference of each measure between graph types at each sparsity. We used brain connectivity toolbox for the estimation of global and local efficiencies and modularity[8].

Fig. 5 in the manuscript showed the results of (a) h_{vol}, (b) h_{fun}, (c) Q, (d) h_{spe}, (e) e_{loc}, and (f) e_{glo}. We plotted the box plots at the sparsity 0.09,0.18,0.27,0.36,0.45, and 0.54 from left to right. In each figure, the horizontal and vertical axes represented the graph type and the network measure, respectively. The color of line was changed by the type of graph: blue for RE, green for SW, red for RA, cyan for SF, and magenta for HY. In Fig. 5 (a) e_{glo}, the order of five graph types was changed four times at the sparsity 0.04,0.12,0.20, and 0.48. The inconsistent order was due to SF and HY, and the order of RE, SW, and RA was comparatively consistent for the sparsity with RE ≤ SW ≤ RA. In (b) e_{loc}, the order of graph types was also changed four times at the sparsity 0.04,0.08,0.40, and 0.66. e_{loc} also consistently discriminated RE, SW, and RA in the order of RA < SW < RE. However, the order with SF and HY was inconsistent. In (c) Q, the order was RA ≤ SF < HY < SW < RE for all sparsity ($p < .001$, FDR-corrected). In (d), h_{fun} of five network types was always the same at the fixed sparsity. It was because the functional entropy consider only the distribution of edge weights, not the network topology. In addition, the functional entropy did not monotonically increase or decrease over sparsity. Thus, it could also not distinguish the difference in the sparsity of graph. In (e) h_{spe}, the order of graph types in h_{spe} was changed six times at the sparsity 0.04,0.12,0.16,0.30,0.40, and 0.62. The order of graph types in h_{spe} highly depended on the sparsity. h_{spe} measured the connectedness of a graph. Because all nodes in RE had the same degree, RE did not have a modular structure and it always had the smallest h_{spe} among all five graph types. In (f) h_{vol}, the order of graph types was consistent for all sparsity. The order was RE < SW < RA < SF ≤ HY ($p < .001$, FDR-corrected). The volume entropy of SF was similar to that of HY at large sparsity. It was because that as the number of edges increased in a graph, SF lost its sparse property. The volume entropy also distinguished well between RE, SW, RA, SF, and HY.

4 Simulation using artificial weighted networks

In this simulation, we compared the six graph measures in discriminating three distinct types of artificial weighted networks. These three types of weighted networks had the same topological structure, but they had different edge weights. We generated 150 hyperbolic unweighted graphs using CNM toolbox, and defined the edge distance of the graph in three different ways,

- Uniform edge distance (U): all edges had the same distance,
- Long edge distance with high node degrees (L): the edge distance was proportional to the degree of its initial and terminal nodes, $i(e)$ and $t(e)$, determined by

$$l(e) = \frac{\log(k_{i(e)} - 1) + \log(k_{t(e)} - 1)}{\sum_{\forall v \in V} k_v \log(k_v - 1)}, \tag{1}$$

  where k_v is the number of edges connecting with a node $v \in V$[7].

- Short edge distance with high node degrees (S): the edge distance was inversely proportional to the degree of two connected nodes, determined by the inverse of $l(e)$ in (1).

These three networks had the same topology, but different geometries. The edge connecting nodes with higher degree was longer in L, but shorter in S. Thus, it could be assumed that the information propagation was the fastest in S, followed by U and L. Note that before estimating the graph measures, we normalized the volume of weighted graph to two, i.e., $\sum_e l(e) = 2$.

The results of graph measures were shown in Fig. 6 in the manuscript. In each figure, three weighted graphs, S, U, and L were represented by red, blue, and green, respectively. In (a) e_{glo}, the order of S, U, and L was changed three times at the sparsity 0.04,0.12, and 0.26. The order of graph types, S, U, and L was consistent for all sparsity in (b) e_{loc}, (c) Q, (e) h_{spe}, and (f) h_{vol}. The order was L < U < S in h_{vol} in Fig. 6 (f), S < U < L in Q and h_{spe} in (c) and (e), and U ≤ S ≤ L in e_{loc} in (b) ($p < .001$, FDR-corrected). In (d), the order of graph types in h_{fun} was changed twice at 0.04, and 0.80. h_{fun} could not find the difference between S and L.

5 Comparison with the previous studies

We estimated the volume entropy, global and local efficiencies, and modularity of the normalized and unnormalized metabolic networks of Y and O based on PET imaging data. We performed the 1000 permutations for the statistical analysis. We estimated the significance of the volume entropy, global and local efficiencies, and modularity in Y and O as well as the significance of the difference of those measures between Y and O. The results are shown in Table 2 in the supplementary material. The volume of normalized and unnormalized networks was not significant in Y and O, and the difference of the volume between Y and O was also not significant both in the normalized and unnormalized networks.

Table 2. p-value of the volume entropy, global and local efficiencies, and modularity of the normalized and unnormalized metabolic networks of Y and O. The p-value of the difference of the four network measures between Y and O are also summarized.

p-value	Group	h_{vol}	e_{glo}	e_{loc}	Q
normalized	Y	0.729	0.651	0.480	0.610
	O	**0.008**	0.268	0.739	0.198
	Diff.	0.069	0.306	0.372	0.281
unnormalized	Y	0.333	0.330	0.367	0.610
	O	0.545	0.700	0.723	0.198
	Diff.	0.400	0.306	0.312	0.281

We estimated the volume entropy, global and local efficiencies, and modularity of the normalized and unnormalized functional networks based on the fMRI data of 38 subjects. We estimated the significance of the relationship between the four measures and the age of subjects. The results are shown in Table 3 in the supplementary material. The volume functional of networks was negatively correlated with age ($r = -0.411, p < .05$).

References

1. Yao, Y. *et al.* The increase of the functional entropy of the human brain with age. *Sci. Reports* **3**, 2853 (2013).

Table 3. Correlation coefficients and their p-values between age and the volume entropy, global and local efficiencies, and modularity of the normalized and unnormalized functional networks.

corr(p-value)	h_{vol}	e_{glo}	e_{loc}	Q
normalized	**-0.456 (0.004)**	-0.283 (0.086)	0.297 (0.070)	**-0.325 (0.046)**
unnormalized	0.176 (0.291)	**0.441 (0.006)**	**0.392 (0.015)**	**-0.325 (0.046)**

2. Sato, J. R., Takahashi, D. Y., Hoexter, M. Q., Massirer, K. B. & Fujita, A. Measuring network's entropy in ADHD: A new approach to investigate neuropsychiatric disorders. *NeuroImage* **77**, 44 – 51 (2013).

3. Bassett, D. S. Small-world brain networks. *Neuroscientist* **12**, 512–523 (2006).

4. Eguiluz, V. M., Chialvo, D. R., Cecchi, G. A., Baliki, M. & Apkarian, A. V. Scale-free brain functional networks. *Phys. Rev. Lett.* **94**, 018102 (2005).

5. Krioukov, D., Papadopoulos, F., Kitsak, M., Vahdat, A. & Boguñá, M. Hyperbolic geometry of complex networks. *Phys. Rev. E* **82**, 036106 (2010).

6. Alanis-Lobato, G. CNM: A a matlab toolbox for the construction of artificial complex networks. https://kr.mathworks.com/matlabcentral/fileexchange/45734-cnm/ (2014). [Online; accessed 03-Mar-2014].

7. Lim, S. Minimal volume entropy on graphs. *Trans. Amer. Math. Soc.* **360**, 5089–5100 (2008).

8. Rubinov, M. & Sporns, O. Complex network measures of brain connectivity: Uses and interpretations. *NeuroImage* **52**, 1059–1069 (2010).
